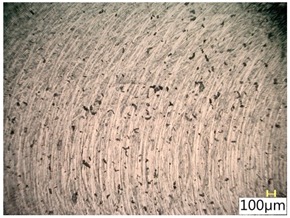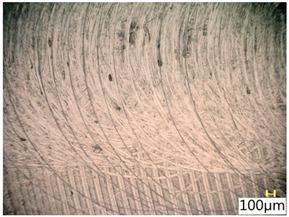# Measurements of Forces and Selected Surface Layer Properties of AW-7075 Aluminum Alloy Used in the Aviation Industry after Abrasive Machining

**DOI:** 10.3390/ma12223707

**Published:** 2019-11-10

**Authors:** Jakub Matuszak, Mariusz Kłonica, Ireneusz Zagórski

**Affiliations:** Department of Production Engineering, Mechanical Faculty, Lublin University of Technology, 20-618 Lublin, Poland; m.klonica@pollub.pl (M.K.); i.zagorski@pollub.pl (I.Z.)

**Keywords:** ceramic brush, force, surface roughness, surface free energy, brushing

## Abstract

Measurements of forces during machining, especially thin-walled structures typical of the aviation industry, are important in the aspect of instability caused by vibration. One of the last stages of manufacturing by machining is the finishing treatment and deburring of the product’s edges. Brushes with ceramic fibres are often employed in deburring, especially for large-sized elements specific to the aviation industry due to the possibility of automatic machining directly on machining centres. This study set out to analyse the effect of variable brushing conditions on axial forces and the selected surface layer properties of AW-7075 aluminium alloy. Experimental studies have examined factors such as surface roughness and topography, axial cutting force in ceramic brush treatment and surface free energy in the aspect of adhesive joints. The tested variable process parameters were the fibre material and the adjustment sleeve spring stiffness. Based on the tests, it was found that the axial force applied by the brush was more strongly connected with the spring stiffness rather than the type of bristle. For most cases, an increase in the value of free surface energy after brushing was observed compared to the initial machining which was milling.

## 1. Introduction

Brushing is a type of surface treatment with a rotary tool. Brushing tools are used in surface treatment processes as well as for deburring, edge rounding, fine shining, and broadly understood surface cleaning. Brushing is found in a number of industries requiring high-quality machined surfaces [1,2]. Analysis of the surface layer [3] of machine parts is frequently the primary subject of scientific studies Deburring is a popular application of brush treatment. An in-depth analysis of the impact of the brush treatment on the efficiency of deburring and the surface finish is presented in numerous studies [4,5,6]. These analyses focused on the state of the edge (in rounding or chamfers), edge roughness, microhardness, and internal stress, and they demonstrated a significant increase in microhardness and inherent stress after brushing. Employing both directions of brush rotation (up and down cutting) along the edges increases the efficiency of deburring [7].

Burr is found at the tool exit from the cutting zone [4]. Given the degree of automation, deburring methods may be classified into manual, semi-automated, and fully automated. This distinction is quite relevant as brushing technology fosters full automation and, thus, may be performed by CNC machines on both metal and non-metal substrates. Brush treatment is suitable for basic applications, i.e., the removal of surface contaminants—including corrosion products and special operations such as surface treatment preceding the deposition of protective/decorative coats or the removal of undesired products from previous treatments—and burr or welding residue.

The effectiveness of using special brushes in deburring the Inconel 718 alloy and the titanium alloy Ti6Al4V following drilling was proven in earlier studies [8]. This study also assessed the effect of brush treatment with five types of brushes of different grain size on the surface roughness of the machined holes. It was shown that the surface of holes drilled in titanium alloy Ti6Al4V and Inconel 718 after brushing exhibited lower roughness and dispersion compared to the untreated surface.

Diamond-abrasive grain tools were proven to be a suitable solution in frosting and other metal treatment operations in a study by Novotný et al. [9], the primary focus of which were the cutting forces generated in the contact zone and the filament deformations over the entire period of contact with the workpiece.

In their work, Rodríguez et al. [10] presented a detailed analysis of the effects of brush treatment on cast-iron brake discs as a potential alternative to grinding. Brushing successfully removed spiral machining marks, resulting from turning, from the surface of brake discs. The machining marks reduce braking efficiency and generate vibration and noise in the initial period of brake operation. Two types of cup brushes were evaluated from the perspective of the impact of technological parameters (a_p_, feed rate) and the number of brush passes on the surface roughness of the test material. It was shown that brushing, compared to grinding, provides a cost-effective and efficient solution for reducing the surface roughness and removing machining marks after turning.

Kitahara et al. [11,12] examined the impact of brush treatment on the microstructure, mechanical properties and corrosion resistance of AZ31B magnesium alloy. The researchers showed that the grain size of the magnesium alloy microstructure in the surface layer after brushing shows higher dependence on feed rather than the clamping force, in the range of the machining conditions adopted in the experiment. The yield strength and tensile strength increased slightly after brushing. The brushed surface had high corrosion resistance, about four to seven times greater compared to the untreated substrate.

The cutting forces and their change during machining are among the key machinability indices, which is why we have already attempted to approach them in our previous scientific works [13]. Analysis of amplitudes in cutting provides the source of data regarding the generation of vibrations during machining, which is essential with respect to thin-walled workpieces [14]. The issues emerging in the machining of thin-walled elements, e.g., the shape and dimensional accuracy, the cutting forces, and the surface roughness, have been extensively described in the literature [15,16,17].

Therefore, the analysis of real cutting force components [18] and vibrations [19,20], which frequently implement computer-generated models (e.g., artificial neural networks), contributes to improving machining stability and efficiency. An important feature of HSM (High Speed Machining) is revealed when operating at increased cutting speeds (constant-volume efficiency of the process)—the cutting force is reduced thus enabling milling of thin-walled elements (e.g., up to 0.1 mm) [21]. Since the dependencies among the factors above are typically non-linear, attempts are made to model machining processes by means of mathematical analytical methods [22].

The concept of automatic lapping of surfaces with ceramic brushes after milling was presented by Kim et al. [23], who analysed in detail the effects of treatment depending on the machining parameters feed, rotational speed, various preloads, and overlapping machining pitch. The study included experimental tests and numerical simulations, which both showed fibre deflection depending on the protrusion. The tests were carried out with brushes, 15 mm in diameter, consisting of 14 fibre bundles, where each bundle contained 50 individual ceramic fibres. The results showed that, for the initial roughness Ra = 2.07 µm, a 10 fold reduction in the surface roughness is possible with appropriately selected brushing treatment parameters. In addition, the results allowed the authors to determine the stroke value among individual passes relative to the brush diameter that produces a uniform surface structure without visible roughness which is conducive to implementing process automation when working on large surfaces.

Computer modelling is a cost- and time-saving tool which reduces the number of iterations in the optimisation of cutting data by reducing the amount of machining error and waste. Moreover, the distribution of cutting forces is of great importance in the aerospace and medical industries, demanding high-accuracy of complex thin-walled elements [24]. Monies et al. [25] tested the performance of plunge milling and BotTCF (balancing of the transversal cutting force) ramping at successive depth levels for producing very deep pockets in magnesium rare-earth alloy MRI301F (Mg-Nd-Y-Zr-Zn) workpieces. In the said study, computer modelling was employed to produce the cutting force components data. Extreme cutting forces significantly reduce the quality of the surface finish as a result of the increased chatter generated in the machine tool system. The results showed that the maximum tangential, radial, and axial cutting forces were for one tooth cutting the material (zone 1): Ft = 1136 N, Fr = 450 N, Fa = 253 N—a resulting force was 1248 N; for two teeth cutting the material (zone 2): Ft = 572 N, Fr = 330 N, Fa = 468 N—a resulting force FRmax was 827 N, whereas the minimum and maximum transversal cutting forces amounted to Fxmax = 364 N.

As the cutting force is, to a great extent, affected by accidental adhesion and built-up edge, the resulting fluctuations could substantially decrease the quality of surface finish and the shape and dimensional accuracy [26] while increasing the temperature in the area of cut [27].

The energy state of the surface layer of modern industrial-grade structural materials (aluminium alloys, magnesium alloys or composites) found, for example, in the aviation industry, is essential in technologies where effectiveness heavily relies on proper adhesion. These technologies include adhesive bonding, sealing, and applying a protective or decorative coating. Adhesive joining of structural materials therefore must be preceded by proper surface preparation of substrates. Burrs typically appear on the edges of machined parts, i.e., the area which is critical to the formation of a strong joint among adherents. Therefore, deburring by brush treatment could constitute the last operation prior to joining, adhesive bonding or coating. Therefore, it is of great importance to verify the surface free energy after brushing in order to decide whether brushing technology is necessary. The preparation of the surface layer of adhered substrates is absolutely critical, particularly in a situation when the bonded assemblies are expected to perform well under variable temperature cycle loading [28,29,30,31].

Ceramic brushes are a relatively recent solution introduced for deburring and surface layer properties modification. By analysing surface roughness, topography, surface free energy, and forces occurring during brushing, the effect of tool on the workpiece can be determined.

## 2. Materials and Methods

### 2.1. Material

The material tested was AW 7075-T651 aluminium alloy which offers good machinability, strength, thermal conductivity, and corrosion resistance. These properties earmark it for use in the production of thin-walled components. The chemical composition and physical properties of the substrate material used in the tests are given in Table 1.

### 2.2. Test Setup

The treatment was performed with the use of a XH712G vertical machining centre and auxiliary equipment.

**Figure 1:**
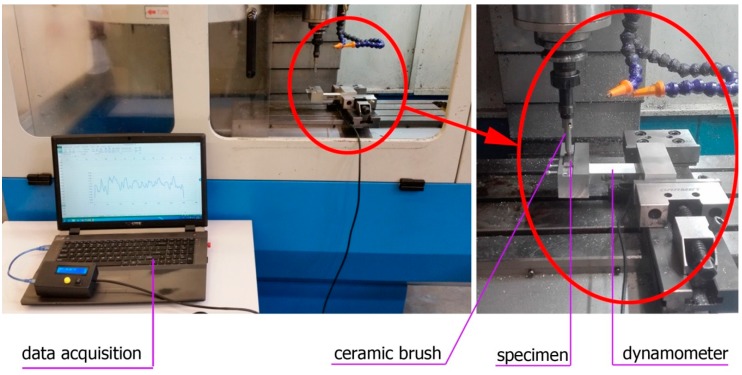
The system for brushing force measurement.

**Figure 2:**
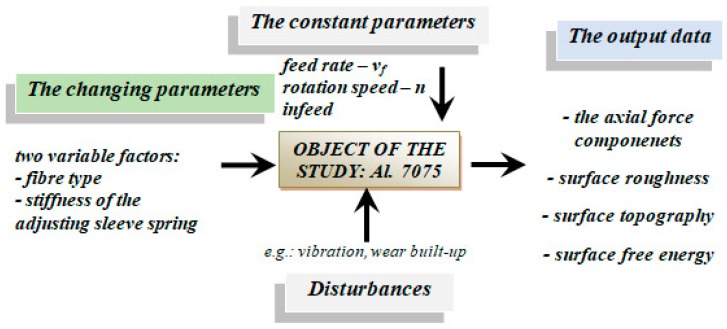
Schematic diagram of the study object.

The readings were carried out by the dynamometer fixed in a machine vice holding the test specimens. The specimens were perpendicular to the brush axis. Figure 1 shows the test setup, while the schematic diagram of the study is shown in Figure 2.

### 2.3. Tools

Prior to ceramic brush treatment, all specimens were subjected to milling with an Iscar solid carbide milling cutter (diameter D = 20 mm) with the tool geometry dedicated for light alloy machining. Constant milling parameters (v_c_ = 500 m/min, f_z_ = 0.1 mm/tooth, a_p_ = 0.5 mm) were employed in order to ensure that the finish of the surfaces exhibited constant roughness. Thus, produced surfaces were analysed to determine the effect of brushing on the value of surface roughness parameters in relation to milling.

**Figure 3:**
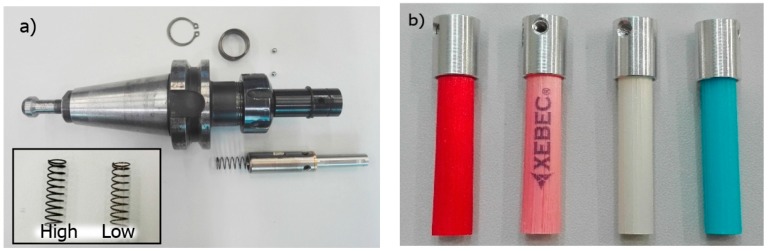
Ceramic brushes: (**a**) brush adjustment sleeve; (**b**) fibre types.

The tested Xebec brushes for deburring applications are shown in Figure 3. In Figure 3a, the sleeve is mounted in a standard tool holder; in addition, the low- and high-stiffness adjustment springs provided with the brush set are shown. The wire thickness of the “LOW” spring was 0.58 mm and the “HIGH” was 0.68 mm.

The colours of the presented brushes indicate different flexibility parameters and diameter of individual fibres:

- Pink – highly flexible, low impact on surfaces;
- Red – flexible, deburring soft materials;
- White – rigid, high efficiency, deburring, reducing surface roughness;
- Blue – extremely rigid, high efficiency in machining difficult-to-cut materials.

### 2.4. Brushing Conditions

**Figure 4:**
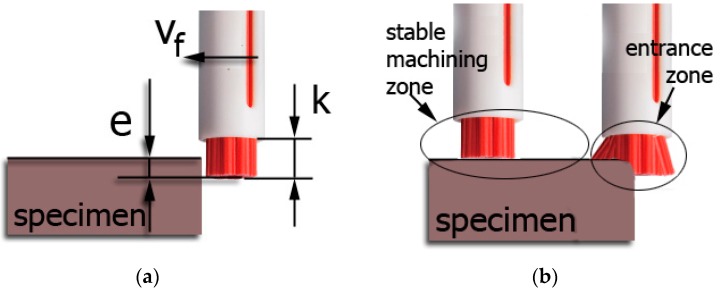
Brushing process: (**a**) graphical representation of infeed “e” and fibre projection length from sleeve “k”; (**b**) machining zone presentation.

In the experimental stage of the study, we undertook to determine the degree the different brushing conditions affected the axial force applied by the brush on the workpiece surface. Specifically, two variable factors were investigated: fibre type and adjustment spring stiffness, while the constant machining parameters were rotation speed n, feed rate vf, and infeed and fibre projection length “k”. Infeed is schematically indicated in Figure 4, and it represents the distance between the fibres and the work surface, i.e., depth of cut.

The brushing data were derived from the manufacturer’s recommendations (Table 2), and the process was performed in five repetitions for each of the cutting data scenarios.

### 2.5. Measurements Methodology

#### 2.5.1. Force Measurement Sensor

**Figure 5:**
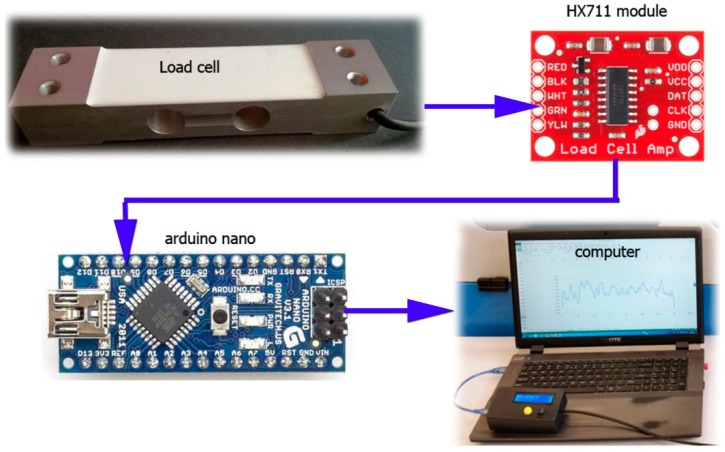
View of the dynamometer components

The cutting forces in the brushing were measured with the use of a custom-built, single-circuit dynamometer shown in Figure 5. The measuring setup consisted of four elements. The loading cell NA1, providing readings of up to 40 kg (400 N) at a measuring accuracy of 0.01 N, was fitted in an aluminium body. Prior to the measurements, the tensometric beam was calibrated by applying a specific load to the beam and introducing a mathematical model transcribed in C++ to a microcontroller, converting readings from the measurement beam into force. The electric signal from the tensometric beam was transmitted to the HX711 load cell amplifier (working at a 10 Hz or 80 Hz frequency) and, subsequently, to an Arduino Nano microcontroller, acquiring and converting the received signal into data. The measurement results were exported to an MS Excel sheet in real time via COM connection and an auxiliary application developed in the Visual Basic environment.

The correctness of the force gauge used was verified against the readings from the 9257B Kistler.

#### 2.5.2. Surface Roughness Measurements

The effect of brush treatment on surface roughness was assessed with the application of 2D and 3D roughness parameters which were measured after brushing. The 2D profile measurements were performed with a Taylor Hobson Surtronic 3+ (Taylor Hobson Ltd., Leicester, UK) (Ra and Rz) whereas the surface topography was measured using a Hommel-Etamic 3D T8000 RC120-400 device (Jena, Germany).

#### 2.5.3. Texture Measurements

The texture measurements after brushing were performed with a Keyence VHX-5000 digital microscope (Osaka, Japan). The results from the measurements provided the data for the assessment of the impact of particular fibre types and brush adjustment sleeves on the intensity of the impact on the machined surface.

#### 2.5.4. Surface Free Energy Measurements

The contact angle measurements of the surfaces under tests were carried out with distilled water and diiodomethane. The measuring liquids with a constant volume of 4 mL were automatically deposited on the test surface with the PGX goniometer (Paul N. Gardner Company, Pompano Beach, FL, USA). Ten measurements of the contact angle were performed for each measuring liquid and on each tested surface. Subsequently, the obtained values were averaged and served to estimate the free surface energy of specimens.

## 3. Results

### 3.1. Axial Force

**Figure 6:**
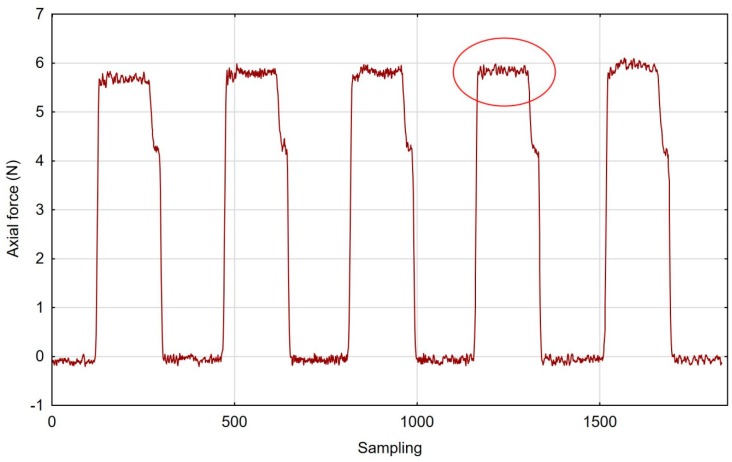
Axial force during brush treatment with the pink fibre tool and “low” spring in the offset adjustment sleeve.

Following five repetitions for each set of cutting data, we obtained the results represented in the graph in Figure 6. Here, the cutting force changed during brushing, and the pink fibre tool and the “low” spring in the offset adjustment sleeve are presented in Figure 6. Moreover, a low scatter of axial forces measured during machining is indicative of good repeatability for the entire process.

By establishing stable machining zones, we managed to filter out the non-machining results.

**Figure 7:**
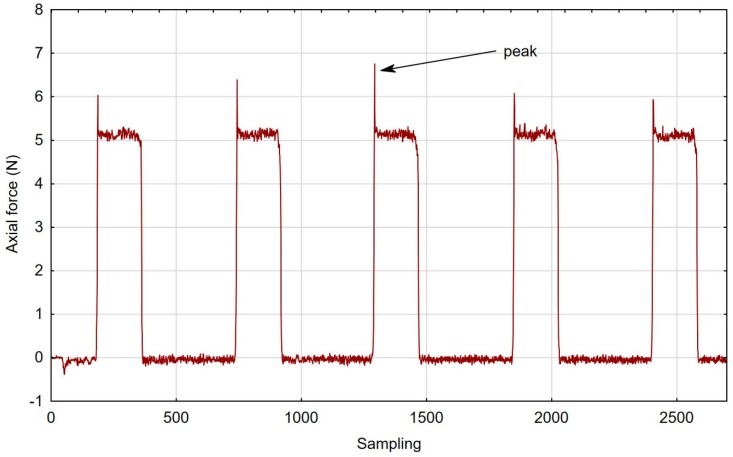
Axial force during brush treatment with the white fibre tool and “low” spring in the offset adjustment sleeve.

Characteristically, the graphs showing characteristics of fibres that were more rigid contained typical cutting force peaks when the tool was entering into the cut (Figure 7).

**Figure 8:**
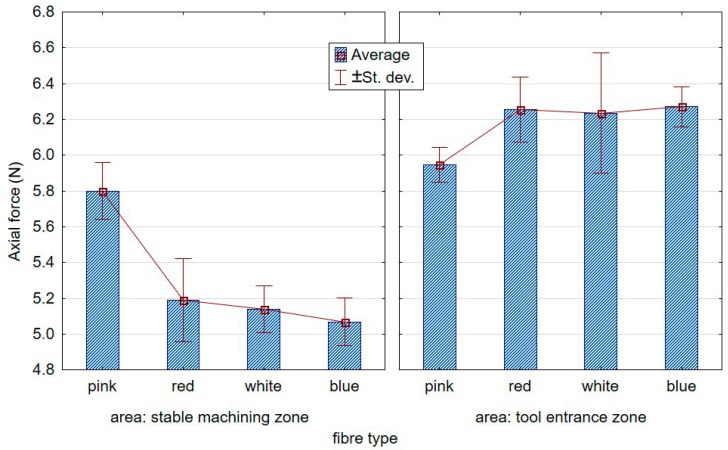
Effect of the fibre type on axial forces at the tool entrance zone and the stable course for the “low” spring in the offset adjustment sleeve.

**Figure 9:**
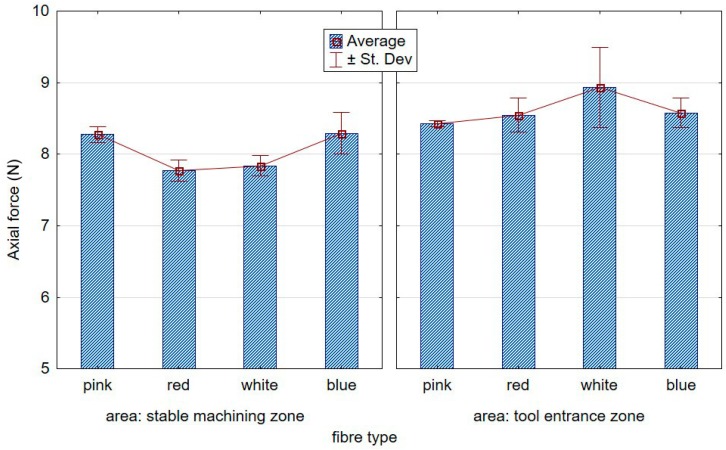
Effect of the fibre type on the axial forces at the tool entrance zone and the stable course for the “high” spring in the offset adjustment sleeve.

The first point of contact of the less flexible brushes was characterised by high dynamics, ergo the peaks in the graphs, as opposed to the less aggressive tools, entered gently into the workpiece. Figure 8; Figure 9, respectively, show the contrasting average results for the low and high stiffness springs over five repetitions. From the graph, a growing distance between the stable machining and the tool entrance zones can be observed for the lower stiffness spring.

**Figure 10:**
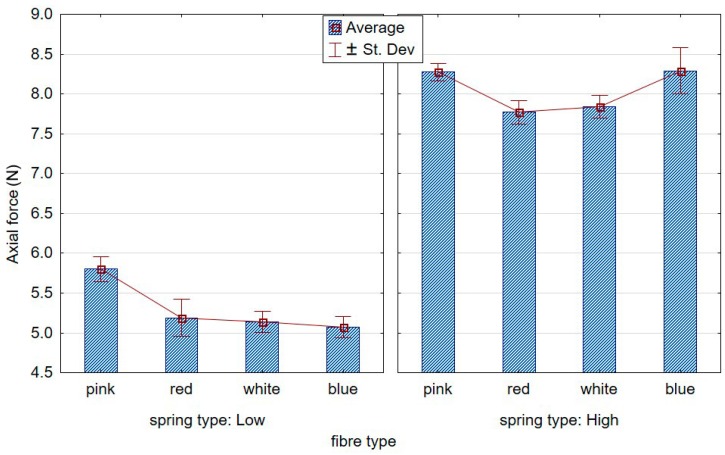
Effect of the fibre type on the axial force in the stable cutting zone for the two tested springs.

Figure 10 compares stable machining axial forces recorded for low and high stiffness springs. A significant increase in the force value was observed when the spring in the brush adjustment sleeve was of higher stiffness. In addition, with respect to “low” spring stiffness, the axial force was seen to decrease as the fibre stiffness increased.

### 3.2. Surface Roughness

**Figure 11:**
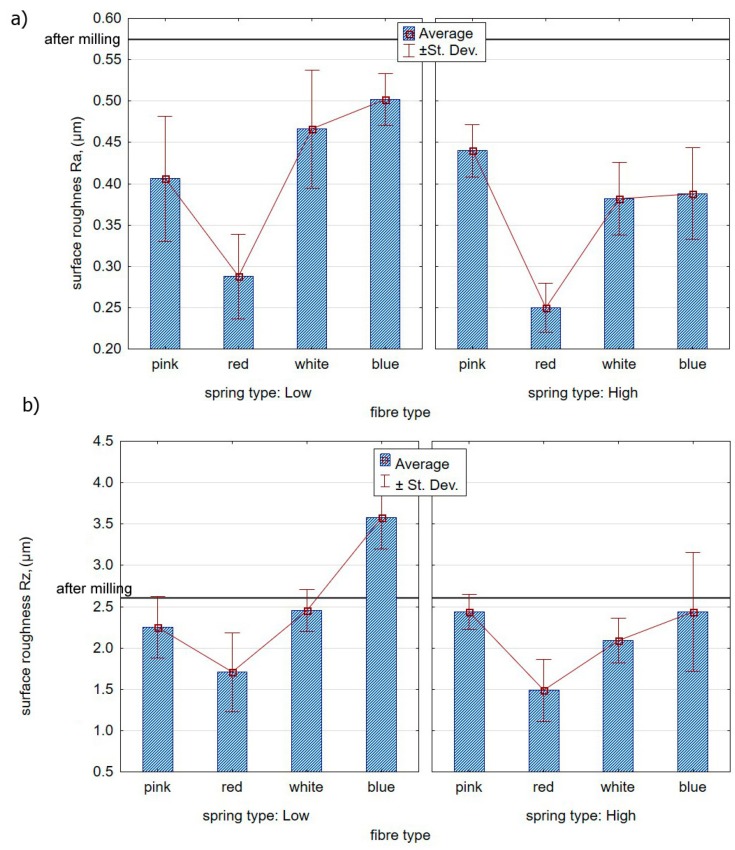
Effect of the fibre type and spring in the offset adjustment sleeve on the surface roughness: (**a**) Ra parameter; (**b**) Rz parameter.

The surface roughness measurements after brush treatment assessed the effectiveness of the process, leading to the reduction of the roughness parameters after milling as a result. Figure 11a presents the impact of ceramic fibre stiffness on the value of the roughness parameter Ra. The results are presented for two springs mounted in the brush adjustment sleeve in Figure 11. The red fibre brush proved to be the most efficient in terms of improving the condition of the surface layer of the specimens following milling—the roughness decreased from the mean Ra = 0.575 after milling to Ra = 0.288 after brushing.

Figure 11b shows the impact of brush fibre stiffness on the Rz parameter. It can be seen that the blue fibres, characterized by the highest stiffness and “low” spring stiffness settings, the value of the Rz roughness parameter increased in relation to the values obtained after milling.

### 3.3. Surface Topography

**Figure 12:**
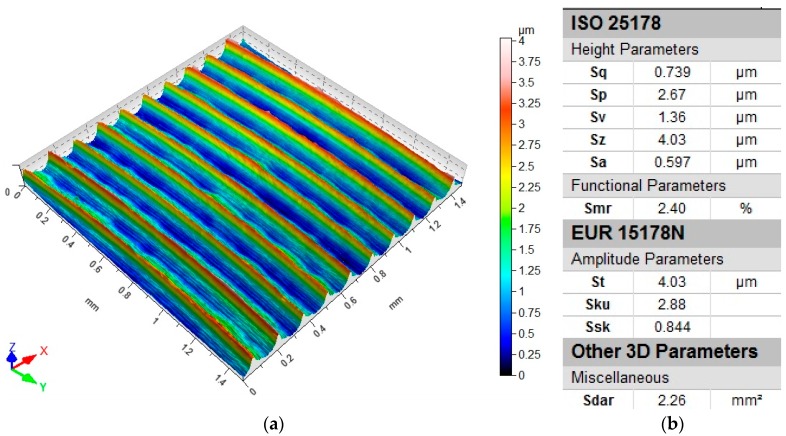
Roughness following milling: (**a**) topography; (**b**) parameters.

Surface topography analysis allows for a more complete analysis compared to 2D parameters. Figure 12 shows an example of surface topography after milling. Characteristic traces are the result of mapping the shape of the blade on the surface, and the intervals of individual traces correspond to the value of feed per tooth f_z_.

Table 3 presents sample maps showing the surface topography after brushing treatment for the “low” spring in the adjustment sleeve. The largest decrease of the Sa parameter in relation to milling was observed for “Red” fibres. This was due to the shear vertices of surface micro-unevenness by the fibres of the rotating brush.

Table 4 presents sample maps showing the surface topography after brushing treatment for the “high” spring in the adjustment sleeve. In the case of fibres with higher stiffness (i.e., white and blue), characteristic scratches are clearly visible on the roughness maps which are the result of more aggressive subtraction of material by the brush fibres, compared to softer fibres (i.e., pink and red). The effect was further confirmed by the increase in the values of the Rz parameter.

### 3.4. Surface Texture

**Figure 13:**
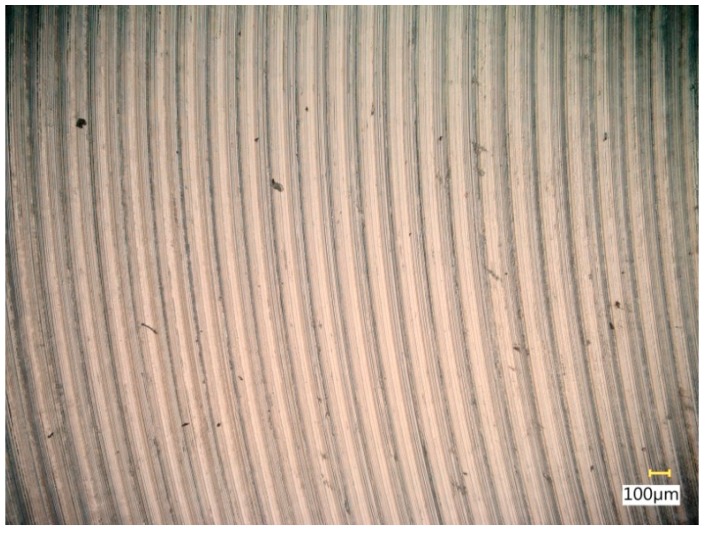
View of the surface after milling.

Figure 13 shows a surface after milling, which clearly shows the periodic pattern of micro-irregularities—a characteristic product of milling. The gaps among the traces correspond to the specified feed per tooth.

Table 5 shows a view of the surface after brushing with the use of the “low” spring in the adjustment sleeve. In the case of brushing with pink and red fibres, a greater efficiency in removing marks after milling was observed. This may be due to the fact that elastic fibres adapting to micro-grooves after milling allow better material removal. In addition, the elasticity of the fibres allow for a greater proportion of contact compared to more rigid white and blue fibres.

Table 6 shows a view of the surface after brushing with the use of the “high” spring in the adjustment sleeve. Characteristic dark spots may indicate that brushing products are on the treated surface.

Considering the blue fibres and the “high” spring stiffness in the brush adjustment sleeve, characteristic grooves on the surface are visible which are typical products of machining with rigid fibres.

### 3.5. Surface Free Energy

**Figure 14:**
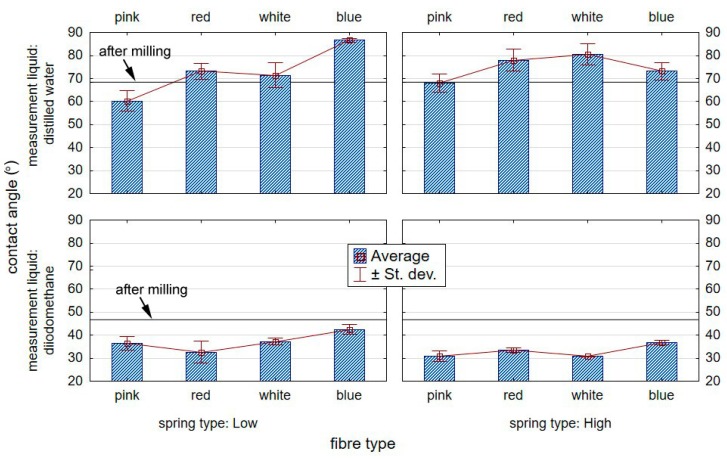
Effect of brushing on the value of the contact angle on the surface of an aluminium alloy.

Figure 14 shows the effect of brush treatment of aluminium alloy specimens on the value of the contact angle with standard liquids.

From the obtained test results, it can be seen that the type of brush employed in treatment has a great effect on the values of the measuring liquid contact angle measured on the surface of aluminium alloy substrates in relation to the specimens prior to brushing. The highest drop in the contact angle for distilled water was recorded for the surface treated with the “pink” fibre brush together with “low” spring stiffness in the brush adjustment sleeve compared to the samples before brushing. The decrease was approximately 12%.

**Figure 15:**
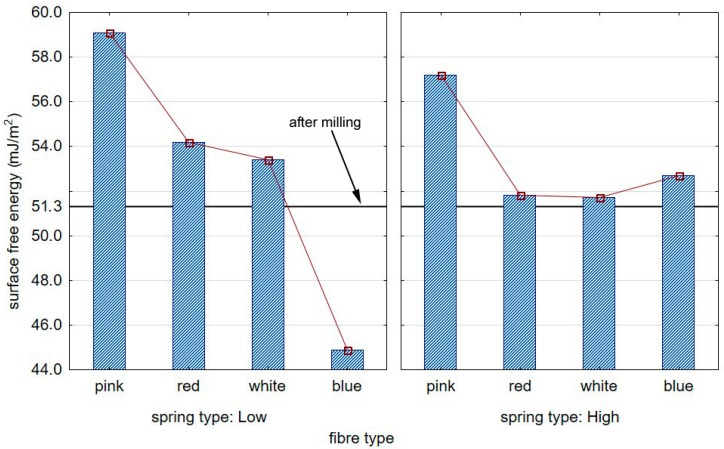
The impact of the brush type on the SFE (surface free energy) of the aluminium alloys.

Figure 15 shows the impact of the type of brush on the value of the surface free energy measure on the surface of aluminium alloy AW7075 specimens.

The most substantial increase in the value of the surface free energy was observed for samples subjected to treatment with a pink-fibre brush and “low” spring stiffness in the adjustment sleeve, which was approximately 15% compared with the specimens prior to brushing.

## 4. Discussion

The aim of the tests on 7075 aluminium alloy was to determine the effect of various cutting conditions of brushing on the values of axial force, surface roughness, and surface free energy. the conclusions are as follows:

- Machining with high-stiffness fibres led to peaks in the axial force applied by the brush entering into the cut (not observed in the case of the most flexible brush—pink); the peaks might indicate the unsuitability of stiff-fibre brushes for thin-walled flexible substrate applications;
- When machining with the low-stiffness spring in the adjustment sleeve, the axial forces were substantially lower with increasing fibre stiffness;
- Brush stiffness affected the axial force in brushing with the high-stiffness spring only to a small degree;
- The tool entry into the cut showed higher standard deviations owing to the highly dynamic character of the process;
- The lowest surface roughness values were obtained after brush treatment with red fibres, for both “low” and “high” spring stiffness settings.
- The use of rigid fibres (i.e., blue and white) may lead to carving micro-grooves on treated surfaces, which results in an increase in the surface roughness parameters (particularly Sz);
- The use of “pink” brushes and “low” spring stiffness settings in the adjustment sleeve resulted in a significant increase in the value of the surface free energy by approximately 15% compared to the samples prior to brushing. This is of paramount importance, particularly in the aviation industry where adhesion technologies—highly sensitive to improper bonding—are employed in adhesive joint assemblies and in various types of protective or decorative coatings;
- The effect of the spring type on axial force was higher than that of the brush fibre stiffness. Nevertheless, it is more relevant for other effects of treatment (surface finish/roughness, the effectiveness of deburring, etc.)

Given the reported results, it must not be forgotten that the forces emerging during brushing with ceramic fibre tools are considerably smaller than in other finishing treatments. Low forces translate to a lower risk of vibration, which is among the key risks in the machining of low-stiffness, thin-walled parts found in various applications in the aviation industry.

## Figures and Tables

**Table 1:** Chemical composition and physical properties of AW 7075-T651 aluminium alloy [2].

Chemical Composition, wt. %		Physical Properties		Specimen
Cu	1.59	Rm MPa	559	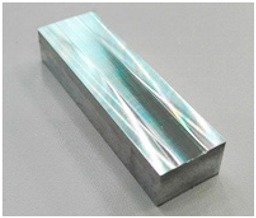
Mn	0.01			
Mg	2.56			
Cr	0.18	Rp_0.2_ MPa	448	
Zn	5.78			
Si	0.07			
Fe	0.13	HB	172	
Ti	0.05			
Al	Rest			

**Table 2:** Brushing conditions.

**Tool Data**			
**Brush Tool**	**Fibre**	**Stiffness of Offset Adjustment Sleeve Spring**	**Fibre Projection Length from Sleeve “k” (mm)**
ceramic brush	pink	high	5.5
		low	
	red	high	
		low	
	white	high	
		low	
	blue	high	
		low	
**Cutting Data**			
**Medium**	**Brush Speed (rev/min)**	**Feed Rate (mm/min)**	**Infeed “e” (mm)**
Dry machining	5000	1000	0.5

**Table 3:** Surface topography after brushing with the “low” spring in the offset adjustment sleeve.

**Pink**	**Red**
Sa = 0.415 µm, Sz = 3.99 µm	Sa = 0.272 µm, Sz = 4.79 µm
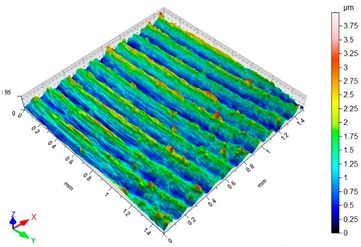	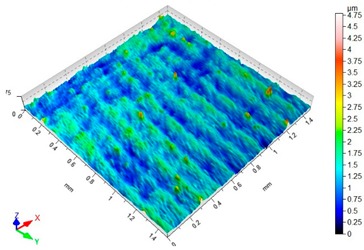
**White**	**Blue**
Sa = 0.394 µm, Sz = 5.69 µm	Sa = 0.336 µm, Sz = 4.57 µm
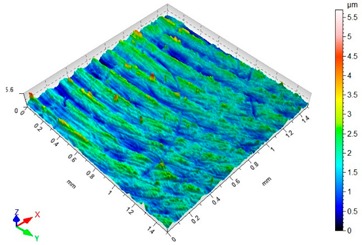	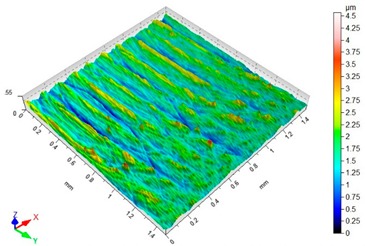

**Table 4:** Surface topography after brushing with the “high” spring in the offset adjustment sleeve.

**Pink**	**Red**
Sa = 0.359 µm, Sz = 5.71 µm	Sa = 0.351 µm, Sz = 5.74 µm
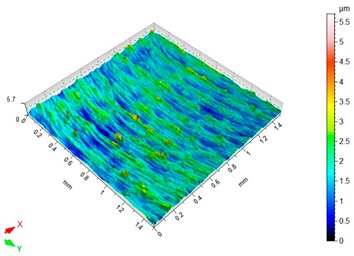	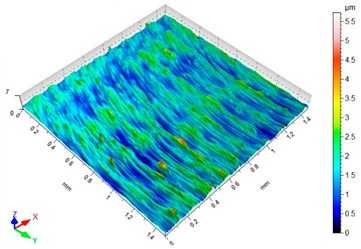
**White**	**Blue**
Sa = 0.338 µm, Sz = 7.08 µm	Sa = 0.312 µm, Sz = 5.73 µm
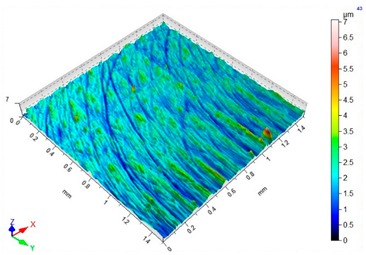	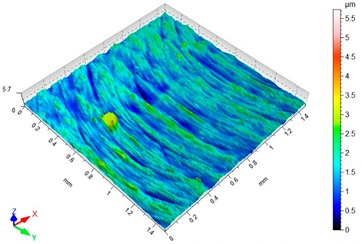

**Table 5:** Surface texture after brushing with the “low” spring in the offset adjustment sleeve.

**Pink**	**Red**
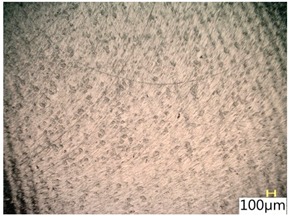	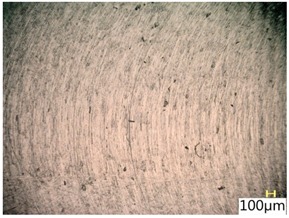
**White**	**Blue**
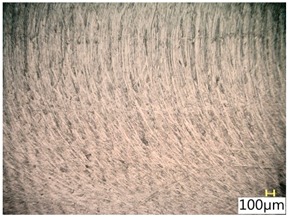	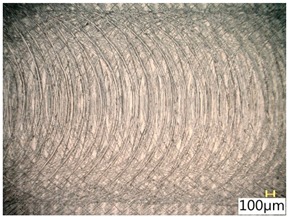

**Table 6:** Surface texture after brushing with the “high” spring in the offset adjustment sleeve.

**Pink**	**Red**
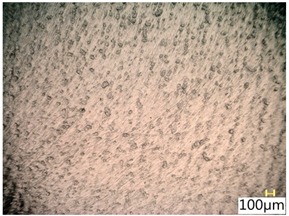	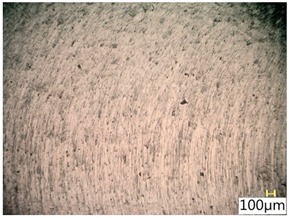
**White**	**Blue**